# Modeling Water Resources: Have We Got it Right?

**DOI:** 10.1100/tsw.2000.5

**Published:** 2001-04-04

**Authors:** Karl E. Havens

**Affiliations:** South Florida Water Management District, West Palm Beach, Florida, 33416-4680, USA

## Abstract

Aquatic scientists generally recognize that controlled experiments are required to establish cause-effect relationships (e.g., Havens and Aumen, 2000), and understanding ecological processes is key to accurately predicting complex ecosystem responses. However, resource managers may have at their disposal only a limited amount of observational data when faced with management decisions. Hence, there may be a tendency to use simple empirical models for decision making. An example of eutrophication management in lakes illustrates a pitfall of this approach when used independently of other scientific information.

For many decades, aquatic scientists have known that blooms of noxious blue-green algae can occur in lakes that are enriched with phosphorus. In lakes undergoing phosphorus enrichment, there often are significant positive relationships between phosphorus inputs, concentrations of phosphorus in water and density of algae. These relationships also are evident in multi-lake data sets, from which many published regression models have been developed. However, a *causal* link between phosphorus inputs and algal blooms was not established by empirical relationships, but through whole-lake nutrient addition experiments (Schindler, 1977) and an understanding of algal nutrient stoichiometry (Hecky and Kilham, 1988). At high rates of enrichment, phosphorus may reach surplus levels, and nitrogen may become the "secondary" limiting factor (Schelske, 1984). Phosphorus-enriched lakes also can develop high rates of internal loading such that water column concentrations become uncoupled from external loading in short time scales (Sas, 1989).

When evaluating a lake with excessive phosphorus loads, one may discover that (1) algal biomass is not correlated with phosphorus and (2) water column phosphorus is not correlated with loads. These findings could lead to a conclusion that algal biomass cannot be controlled by reducing phosphorus inputs. That conclusion often would be incorrect.

In the same way that correlation does not always reflect cause, lack of correlation does not rule it out. Experience has shown that phosphorus-enriched lakes do respond to load reduction, albeit over longer time scales than lakes that are less impacted. Phosphorus load reduction is a necessary element of restoration even when other management actions are required (Moss et al., 1997). Once a lake reaches equilibrium with reduced loads, water column concentrations decline, and when phosphorus no longer is in surplus, algal biomass again becomes dependent on phosphorus supply. No part of this complex response sequence can be predicted simply from empirical relationships at the start of the restoration process.

The example underscores two points. First, strict reliance on empirical models as evidence that causal relationships do or do not exist can lead to false conclusions and incorrect actions. Second, there is a need for close cooperation between scientists and managers, so that a full suite of research tools (observation, experiment, model) can be brought to bear on important environmental issues. These points are particularly important today, when most of the world's fresh waters are impacted by some form of human-related stress.
